# New viruses on the rise: a One Health and ecosystem-based perspective on emerging viruses

**DOI:** 10.2217/fvl-2021-0215

**Published:** 2021-10-14

**Authors:** Koray Ergünay

**Affiliations:** ^1^Department of Medical Microbiology, Virology Unit, Hacettepe University, Faculty of Medicine, Ankara, 06100, Turkey

**Keywords:** ecosystem, emerging, global, novel, one health, virus

## Abstract

Empowered by interdisciplinary collaboration, we now have the tools to identify new viruses, contain future outbreaks and broadly understand natural processes toward a global health.

## Viruses from the laboratory called nature

Never before has virology been on the public spotlight on such a prominent and global level. The ongoing pandemic, caused by the novel SARS-CoV-2, with its dire consequences for almost every country, remains as an international public health emergency. With the example of SARS-CoV-2, new and emerging viruses, their origin, genetic diversity, host range, diagnosis and prevention methodologies have attracted unprecedented interest, sometimes entailing pseudoscientific interpretations extending to conspiracy theories. Meanwhile, the field of virology has already been going through a transformation phase at least for a decade, in terms of overall capacity to identify and characterize new viruses and understanding the biodiversity of the virus world, also termed as the virosphere [1]. The availability and advances in metagenomic sequencing has enabled identification of novel viruses almost on a steady pace, radically altering previous conceptions of the diversity, abundance and structure of the virosphere. Simply put, virology is said to have entered an exciting new discovery phase [1].

## How do we know about viruses? A brief history

Basic concepts of microbiology tell us that all living cells are prone to viral infections and viruses capable of replicating in all known types of prokaryotic and eukaryotic cells, living as single-celled or multicellular organisms, are likely to exist. However, since the advent of virology as a separate field in the realm of microbiology, the majority of the well-characterized viruses have been those involved in human disease or animals and plant infections with significant public health or economical impact. This was partly due to the methodological constraints inherent to then available virus characterization and diagnostic techniques, relying mainly on virus isolation with subsequent cultivation using cell lines or susceptible animals and culture-obtained virus antigens to assess immune responses. Another widely acclaimed notion considered most, if not all, viruses as pathogenic entities, which was in stark contrast with the expected abundance of viruses in the biosphere.

Major advancements for understanding viruses and their infections were provided by DNA sequencing and recombinant DNA technologies that enable antigen production from unculturable viruses. Nevertheless, the most prominent breakthrough was fueled by the dissemination and adaptation of nucleic acid amplification techniques, mostly based on PCR, facilitating a timely detection of ongoing infections as well as virus quantitation. These approaches, despite significant technical advances and automatization, still constitute the mainstay of the current diagnostic virology practices. Prior to the development of metagenomic sequencing techniques as we know today, particular approaches such as consensus PCR, representational difference analysis or sequence-independent single-primer amplification were available and have been instrumental in identifying many novel viruses, virus induced infections and diversity [1–3].

## Metagenomics revolutionizing virus discovery

The impact of metagenomic sequencing on new virus discovery is that it enables relatively sensitive and massively parallel production of target-independent sequences [1]. Despite its obvious advantages over previous approaches, metagenomic sequencing also has particular limitations where optimization of methods and technical improvements will greatly enhance virus discovery. Frequently, the specimens used for the analysis possess minute amounts of the novel virus in question with a huge background of nucleic acids from the host and its microbiome. Therefore, some form of target enrichment or background depletion is commonly employed in virome investigations or efforts for novel virus detection [1,2]. The target virus genome, being DNA or RNA, its amount and purity, further affects the success of the investigation, along with the employed platform and the sequencing approach. Finally, the raw data produced by the sequencing run require particular, usually optimized bioinformatic pipelines and softwares to detect novel viruses. As these pipelines depend on detecting homology between the obtained sequences and those deposited in various databases, divergent viruses distantly related to the known families are harder to identify. Although there is still much room for improvements and optimization according to target and specimen types, metagenomic sequencing approaches have significantly expanded the information and our understanding of the virosphere.

## Expanding on the virosphere

The description of novel viruses and the expansion of the virosphere by the metagenomic approaches come with particular shortcomings and present new challenges. First, this vast array of viruses must be properly categorized and integrated into the existing classification schemes. Traditional virus taxonomy heavily relies on phenotypic properties such as virion morphology, genome type and replication, while phylogenetic assessments provide a better guide to evolutionary relationships. Currently, genomes of the uncultivated viruses outnumber those from virus isolates many times, where phenotypic data are mostly lacking. To facilitate reporting and comparisons, the minimum information about an uncultivated virus genome standards were developed within the Genomic Standards Consortium framework [4]. These standards include reporting of the sequence quality, origin, functional annotation, taxonomic classification, biogeographic distribution of the virus genome as well as *in silico* host prediction. Incorporation of the virus genome data into the official classification scheme of the International Committee on Taxonomy of Viruses has already been under debate and analysis pipelines efficient in predicting assignments according to the current taxonomy framework are being developed [5]. For uncultivated viruses detected via metagenomics, determination of the biological properties poses another colossal challenge. Experiments to investigate cell lines likely to support viral replication should be performed, supported by *in silico* predictions of viral protein structure and function, genome replication and host range. The origin of the virus genome should also be thoroughly questioned, as it may be derived from the initial host, as well as any infecting bacteria, fungus or parasite or from the environment.

Initial clues of the abundance and diversity of the virosphere came from bacteriophages and marine environments [1,6]. Then, studies of life in extreme environments and terrestrial organisms further continued to reveal a surprising diversity and multitude of viruses with several novel strains as well as satellite viruses and subviral agents in plants, vertebrates and invertebrates [7,8]. These findings indicate that our previous conceptions on the virosphere were significantly underestimating the natural diversity and were highly biased, with a broad spectrum of viruses and related genomes indeed being present in every environment and type of organism investigated. A particularly rich spectrum of RNA viruses, dominating the eukaryotic virome and reaching an enormous diversity in animals and plants was observed. They not only filled major gaps in the evolutionary history of RNA viruses but showed an extensive potential for genetic exchange among diverse viruses as well as horizontal transfer between distantly related hosts [8,9]. These findings have important implications for novel viruses pathogenic for vertebrates and their emergence in susceptible host populations.

## Is it possible to predict the next emergent virus?

Emerging infections pose a significant global public health threat and challenge biological and economical welfare in both developed and developing countries. Majority of the emerging viruses are of zoonotic origin, transferred between vertebrates and humans [10]. The term spillover describes the transmission of a pathogen from a natural animal host to a novel host leading to infection [11]. It may occur spontaneously by chance, by novel or repeated exposure and may involve particular genetic adaptive changes in the virus. Driven by the broad-scale factors such as climate change, global transportation and environmental disruption, viruses now have new opportunities to spillover to new hosts and cause epidemic disease. Over the last decades, many examples of high-profile viral infections including HIV, SARS and MERS-CoV, Ebola and Zika viruses have emerged in human populations, the latest and most prominent being the SARS-CoV-2. With the growing body of literature devoted to deciphering the determinants of disease emergence, the extent of the virosphere and virus diversity has also gained additional importance. It is estimated that despite ever-growing number of novel viruses discovered by metagenomics, a vast proportion, over 99%, of the virosphere remains unexplored, with unknown capacity for spillover and pathogenicity [10]. Therefore, the idea of a systematical approach to gradually build an atlas of viruses, with information on sequences, geographical ranges and host distributions comes forward, which is expected to identify those likely to evolve as emergents. Global Virome Project is an initiative based on this idea, conceived to characterize majority of the zoonotic viruses within a decade, to better predict, prevent and respond to future viral pandemic threats [10].

However, it is a hardly attainable task to predict prospectively the emergence of a particular virus, as virologic and epidemiologic factors with very different dynamics and time scales intermingle in the process [12]. Obviously, spillover and cross-species transmission events between animals and humans require constant monitorization. However, understanding virus sharing pathways and spread through an entire spectrum of host species is also a formidable challenge. Nevertheless, network and big data analyses have identified domestic animals as being central in mammalian host–virus interaction and differential spread patterns of DNA and RNA viruses among mammalian groups [13]. Virus sharing among hosts and zoonotic impact are observed to be prevalent for RNA viruses, which also exhibit diminished functional host specificity and potential to shift across hosts within different ecological niches. Metagenomic surveillance in human–animal interface and zones of ecological disturbance also appear as a practical approach to detect and contain the upcoming emerging virus [12]. Hotspots for monitorization would include all sorts of occupational exposure, locations with major changes in land use, live animal markets, locations of regular hunting and butchering of wild animals and areas affected from human population mobility and displacements. Machine learning algorithms are also likely to facilitate processing of the complex bioecological information and develop practicable models regarding transmission [14]. For instance, novel viral genome sequences can be used to predict animal reservoirs and probable arthropod vectors for a diverse selection of RNA viruses [12]. For a better understanding of the virus exposure among susceptible hosts, metaserologic techniques capable of detecting exposure and immune response to a wide range of viruses must be developed and the available assays should be more frequently used for surveillance.

## A peek into the social life of viruses

Viruses are often viewed as hijackers, lone-wolf pirates trying to take over the huge cellular machinery to be exploited for self-replication. However, they do have social and private lives, into which we now have the tools to peek. Virus–virus interactions not only affect the diversity and distribution of viruses but shape the way they interact with the hosts, their environment and ultimately, the ecosystem they reside in [7]. Metagenomics have greatly expanded our knowledge on virus interactions and coinfections which appear as a prominent driver of viral evolution. Inter-virus interactions may result in alteration in infectivity, replication and persistence during concurrent or sequential infection of the host and involve cooperation, communication and competition [15]. Interference and superinfection exclusion, the protection against subsequent infection by a related virus, are well-known outcomes of a possible competition [16]. The interaction between helper-dependent viruses and their helper viruses demonstrates a delicate balance of cooperation and conflict. Defective interfering particles harbor major deletions and require the presence of a functional wild-type virus for propagation. In some viral infections such as influenza, they are observed to affect pathogenicity and host immune response [17]. Cooperation between viruses is also well documented, where independently replicating homologous viruses undergo genetic or phenotypic recombination events during coinfections, producing hybrid particles with enhanced fitness. Moreover, viruses have been shown to disperse in groups among cells or hosts during the infection process [18]. Termed as collective infectious units, they can deliver multiple viral genomes inside the virion or multiple virions inside a larger, vesicle-like structure, with many implications for evolution and pathogenesis. Hence, a better understanding of the virus interactions during infection will surely improve the currently available intervention methods and likely facilitate novel approaches for therapy and control. Metagenomic or multi-omic techniques modified to investigate individual particles or cells greatly enhance the study of virus–virus interactions inside the host.

Virus–virus interactions have particular impact and implications for potential emergent viruses transmitted by arthropod vectors. The natural virome of some invertebrate species, including mosquitoes and ticks, prominent vectors of arthropod-borne diseases, are observed to be immense, with highly-abundant RNA viruses in diverse configurations [1,9]. This diversity and abundance of RNA structures provide unique opportunities for genetic exchange and potential emergence of novel pathogenic viruses, which indicate invertebrates as good candidates for metagenomic surveillance and virus discovery. Due to their interactions with both vertebrates and plants, arthropods also provide a suitable conduit for viruses to move between distinct types of hosts [8]. Interestingly, some of the invertebrate viruses not associated with vertebrate disease are shown to block the replication and diminish total abundance of particular pathogenic viruses in vector hosts, serving to decrease vectorial capacity significantly [19]. This aspect of the interactions among RNA viruses in hosts opens up new possibilities for disease control and warrants further research.

## Toward a unified view of the virosphere within ecosystems

The traditional approach in microbiology tends to isolate components of an environmental niche and tries to provide individual explanations. However, the current accumulation of information requires a more holistic perspective, especially on viruses and their dispersal, spillover and potential emergence. The globally acclaimed ‘One Health’ approach is an effort to establish interdisciplinary collaborations, to better elucidate the underlying mechanisms of many important public health issues, including virus-induced diseases [20]. The need for a multilayered approach has never become so evident in basic and applied virology, to assess virus–virus, virus–host or host–environment interactions, all contributing to disease emergence. It is hard to interpret the impact of bacterial microbiota in health and disease without considering the gut virome, mainly composed of different groups of bacteriophages [3]. We are now beginning to appreciate the vast diversity of viruses in various, sometimes extreme, environments. Fortunately, powerful and robust tools are gradually becoming available to explore viruses in an ecological framework, including interactions with abiotic factors. The contributions of the advancing high-throughput multi-omics technologies, *in silico* analysis tools and machine learning algorithms will intensify and provide crucial insights. As new microbial threats are expected to emerge at an accelerating rate due to the current global conditions, these will be invaluable tools to contain future epidemics and broadly understand natural processes toward a global health.
